# Quiet and Poised: “Silent” Genes Accumulate Transcription Machinery

**DOI:** 10.1371/journal.pbio.1000269

**Published:** 2010-01-05

**Authors:** Richard Robinson

**Affiliations:** Freelance Science Writer, Sherborn, Massachusetts, United States of America

Gene activation—the process of waking up a silent gene and transcribing its DNA—requires many coordinated processes: the gene must be exposed to transcription factors, which must then pile onto specialized sequences adjacent to the gene-called enhancer and promoter regions, which then attract RNA polymerase (the enzyme that catalyzes the synthesis of messenger RNA), which can then attach and prepare to read the gene's sequence.

Within the nucleus, each chromosome sits in its own well-defined domain, called its territory. It has long been observed that activated genes relocate outside their chromosome's territory during expression, leading to the idea that this movement promotes activation by exposing the gene to the transcription machinery.

But is the opposite true? Do genes that remain in their territories have less access to RNA polymerase? It had been thought so, but a new study by Carmelo Ferrai, Sheila Xie, Ana Pombo, Massimo P. Crippa, and colleagues shows that even while sitting quietly within their home turf, some genes are already primed with the transcriptional machinery, poised and ready to go.

The authors used a model gene, urokinase-type plasminogen activator (uPA), which sits on Chromosome 10 and can be chemically induced to increase its expression (mimicking its natural activators, such as hormones). They start by showing that in their inactive state, most of the uPA genes within a population of cells sit within the Chromosome 10 territory, and, upon activation, the majority of them relocate to the outside and transcription goes up. This matches the standard model.

But the authors asked whether this relocation was accompanied by changes in chromatin structure. This would be expected, since opening up the local structure (through changes to the histone proteins upon which DNA is spooled) would allow easier access of the transcription machinery. But, they found that even before the gene was chemically induced to turn on, the regulatory regions of the uPA gene were already open, and that the gene's promoter had already bound the RNA polymerase transcription complex (called a “transcription factory”). In short, the factories had easy access to the gene even when it was buried in the interior of the territory.

RNA polymerase has two critical phosphorylation sites that control its activity, both involving the amino acid serine. In full-on transcription mode, both serine-2 and serine-5 are phosphorylated. When only ser-5 is phosphorylated, the polymerase is “poised,” inactive but ready to go. The authors found that before activation, most of the uPA genes within the Chromosome 10 territory bound poised polymerases. Once chemically activated, the gene preferentially bound the fully active polymerase, along with moving out of the territory. Surprisingly, the interior of the territory was also accessible to fully active polymerase, although there was little transcription there prior to activation.

Finally, the authors measured the transcription frequency both inside and outside the territory after chemical activation. They found that after activation, the frequency with which uPA alleles are transcribed was the same whether the gene was inside or outside, again in contrast to the standard model. Before activation was a different story, though. Genes inside the territory were largely silent, while those few on the outside were transcribed at a rate equal to that of an active gene.

The picture that emerges is both more complicated and more dynamic than the simple inside-quiet/outside-active model of gene activity. At least for this gene, and presumably for many others similarly regulated, the gene is routinely available for binding of transcription factories while still within its chromosome territory. While most of these are poised, rather than active, factories, some transcription does occur there. Upon activation, the gene does move outside its territory, but not to begin the process of accumulating the transcription machinery—this experiment conclusively shows that accumulation of the transcription machinery can and does occur independently and prior to activation. Instead, translocation to the exterior has no influence on the association of induced genes with active factories or a high level of transcription. It is in the uninduced state that the positioning at the CT interior has a silencing effect, The authors suggest that the ability of regulatable genes to be primed with transcription factories while still silent likely allows a more rapid response to environmental signals that turn the genes on.

**Ferrai C, Xie SQ, Luraghi P, Munari D, Ramirez F, et al. (2010) Poised Transcription Factories Prime Silent uPA Gene Prior to Activation. doi:10.1371/journal.pbio.1000270**

